# Biochemical parameters as a tool to assess the nutritional status of patients with amyotrophic lateral sclerosis

**DOI:** 10.3389/fneur.2023.1258224

**Published:** 2024-01-15

**Authors:** Dimitar Monov, Natalia Molodozhnikova

**Affiliations:** ^1^Department of Anesthesiology and Intensive Care, Medical University of Sofia, Sofia, Bulgaria; ^2^Department of Biology and General Genetics, I.M. Sechenov First Moscow State Medical University, Moscow, Russia

**Keywords:** amyotrophic lateral sclerosis, nutritional status, prognostic nutritional index, malnutrition, blood biochemical parameters

## Abstract

**Background:**

The research aimed to analyze blood biochemical parameters in patients with amyotrophic lateral sclerosis and to determine whether they can be used to assess their nutritional status.

**Methods:**

The study included 45 patients diagnosed with amyotrophic lateral sclerosis (ALS): 28 (62.2%) were men and 17 (37.8%) were women. The mean age of the study participants was 50.69 ± 7.24 years. The control group consisted of 30 practically healthy individuals.

**Results:**

Compared with practically healthy individuals, patients with ALS had significantly lower blood parameters, including total lymphocyte count (1.49 ± 0.11 vs. 2.86 ± 0.25, *p* < 0.05), total protein (60.55 ± 2.38 vs. 77.80 ± 4.41, *p* < 0.05), albumin (33.70 ± 2.03 vs. 46.49 ± 3.22, *p* < 0.05), urea (3.09 ± 0.36 vs. 5.37 ± 0.50, *p* < 0.05), creatinine (51.28 ± 4.42 vs. 70.91 ± 5.13, *p* < 0.05), and transferrin (1.84 ± 0.12 vs. 2.32 ± 0.10, *p* < 0.05). These parameters correspond to first-degree malnutrition. There were direct correlations between anthropometric and biochemical parameters in the ALS group. BMI correlated with the blood levels of total protein (*r* = 0.22, *p* < 0.05), albumin (*r* = 0.27, *p* < 0.05), urea (*r* = 0.33, *p* < 0.05), creatinine (*r* = 0.30, *p* < 0.05), transferrin (*r* = 0.18, *p* < 0.05), and total lymphocyte count (*r* = 0.20, *p* < 0.05). PNI correlated with the blood levels of total protein (*r* = 0.53, *p* < 0.05), albumin (*r* = 0.87, *p* < 0.05), total cholesterol (*r* = 0.34, *p* < 0.05), transferrin (*r* = 0.40, *p* < 0.05), total lymphocyte count (*r* = 0.79, *p* < 0.05), urea (*r* = 0, 37, *p* < 0.05), and creatinine (*r* = 0.32, *p* < 0.05).

**Conclusion:**

The study presents compelling evidence supporting the utilization of biochemical parameters, including total protein, albumin, urea, creatinine, transferrin, and total lymphocyte count, for potentially evaluating the nutritional status of individuals diagnosed with ALS.

## 1 Introduction

Amyotrophic lateral sclerosis (ALS) is a progressive, fatal neurodegenerative disease that primarily affects the motor neurons of the central nervous system (1, 2). It poses a pressing challenge in modern medicine due to its disabling nature and the absence of a cure (3, 4). The global incidence of ALS ranges from 4 to 8 new cases per 100,000 people, with a higher prevalence in economically developed countries (4). In regions like the USA and Europe, the annual number of new ALS cases is estimated to be 1 to 2 per 100,000 individuals (1, 5). The majority of patients with ALS succumb to the disease within 3–5 years after the onset of symptoms (1, 3). ALS also imposes a substantial economic burden, exemplified by the annual cost per patient reaching AUD$ 1.1 million in Australia in 2015 (6). Similarly, in Germany, the average annual cost per ALS patient is €78.3 million, with a lifetime cost estimated at €246.1 million, contributing to a total burden of €519.8 million (7).

It is believed that individuals engaged in high-intensity intellectual activities and athletes are at a higher risk of developing ALS (8, 9). Furthermore, the disease exhibits a male predominance, occurring 1.6 times more frequently in men than in women (2). Other identified risk factors for ALS include head injuries, electrical burns, and exposure to heavy metals (9, 10). Genetic factors also play a substantial role, with familial cases comprising 5–10% of all ALS patients and involving more than 25 genes (9, 11).

The pathogenesis of ALS is complex and involves various factors, including genetic predisposition, excitotoxicity, oxidative stress, impaired autophagy, protein aggregation, mitochondrial dysfunction, RNA post-transcriptional modifications, axonal transport disturbances, and neuroinflammation (11–13). Recent studies indicate that ALS can be classified as a proteinopathy, where the central mechanism of motor neuron damage is the formation and aggregation of protein deposits. Initially, this process involves mutant proteins (C9orf72, TDP-43, FUS, SOD-1, senataxin, alsin, etc.), but it later extends to unaffected cellular proteins (14, 15).

As motor neurons degenerate and ultimately die, immune mechanisms become disrupted, leading to a progressive inflammatory response within the nervous tissue (16). Initially, immune cells such as macrophages, T-cells, and glial cells attempt to clear debris from motor neurons and exert neuroprotective effects (16, 17). However, as the disease progresses, these neuroprotective immune-mediated mechanisms weaken, giving way to immune-mediated cytotoxic mechanisms. This shift is accompanied by the release of pro-inflammatory cytokines (IL-1, IL-6, and TNF-α) and neurotoxic molecules (adhesion molecules, proteases, and chemokines), creating a pro-inflammatory environment that exacerbates motor neuron damage (13, 17).

Excitotoxicity is another prominent feature of ALS pathology (18–20). Excessive activation of ionotropic glutamate receptors leads to the influx of excess Ca2+ ions into nerve cells. This influx, in turn, results in the aberrant production of singlet oxygen, endoplasmic reticulum stress, mitochondrial dysfunction, and, ultimately, programmed or premature nerve cell death (18, 20).

Among the early clinical manifestations of motor neuron damage in ALS is the loss of dexterity, impairing patients’ ability to perform motor tasks (5, 21). This impairment is often accompanied by muscle weakness and, in some cases, hyperreflexia or exaggerated reflex responses known as ‘segmental reflexes.’ Over time, these symptoms may progress to paralysis (5, 21, 22). As peripheral motor neurons succumb to the disease, patients may experience paresis, fasciculations, hyporeflexia, and skeletal muscle atrophy, particularly in extensor muscles. Paresis tends to manifest locally, initially affecting either upper or lower extremities and gradually spreading to adjacent muscle groups, often with asymmetry (23). Additionally, bulbar and pseudobulbar syndromes frequently occur, presenting with symptoms such as dysarthria, dysphonia, tongue fasciculations and atrophy, dysphagia, excessive oral secretions, and uncontrollable episodes of crying and laughter (22–24). ALS is characterized by its rapid progression, clinical symptom variability, and association with respiratory and swallowing difficulties, as well as malnutrition (21, 22).

Given that ALS often leads to dysphagia and nutritional status disturbances, early detection of nutritional changes in diagnosed patients is crucial (25, 26). The assessment methods should be both simple and informative, with a focus on both anthropometric and biochemical indicators. This study aimed to evaluate blood biochemical parameters in patients with amyotrophic lateral sclerosis and their potential for assessing the nutritional status of these individuals.

This study posits that biochemical blood parameters, including total protein, albumin, urea, creatinine, transferrin, and overall lymphocyte count, may serve as an effective tool for assessing the nutritional status of patients with amyotrophic lateral sclerosis (ALS). It is hypothesized that alterations in these biochemical indicators may indicate nutritional disturbances in individuals with ALS.

## 2 Materials and methods

The study took place between February 2017 and December 2022. Participants were divided into treatment and control groups (Table 1).

**Table 1:** Characteristics of patient groups.

Group	Number of participants	Gender	Average age (years)
Patients group	45	Men—28 (62.2%)	50.69 ± 7.24
		Women—17 (37.8%)	
Control group	30	Men—19	49.5 ± 6.9
		Women—11	

The treatment group comprised patients diagnosed with ALS. The control group of “practically healthy” individuals was composed to match the age and gender distribution of the amyotrophic lateral sclerosis (ALS) patient group. It included individuals devoid of significant medical comorbidities. These characteristics of the control group were considered to ensure the most comparable baseline conditions with the ALS patient group, enabling a more precise comparison of results and facilitating more informative conclusions regarding the impact of the disease on blood biochemical parameters.

The two groups were within a similar age range. The inclusion criteria consist of individuals between the ages of 18 and 65 years, diagnosed with ALS, and who signed a voluntary informed consent to participate in research. Subjects were excluded from the study if they had the 4B stage of ALS or either of the following conditions: any demyelinating, neurodegenerative disease, dementia, mental illness, cancer, acute or chronic somatic disease (exacerbated or sub−/decompensated), and acute or exacerbated chronic infectious disease. Pregnant and lactating women as well as individuals with alcohol or drug abuse were also excluded and so were subjects who failed to comply with the research protocol.

The ALS diagnosis was made according to the revised El Escorial Criteria (27), patient complaints, and data from neurological examination, electroneuromyography (ENMG), and magnetic resonance imaging (MRI). The neurological examination was performed using standard methods. Damage to the central motor neuron (UMN) was confirmed if the following were present: pyramidal manifestations (deep reflexes), upper paresis, abnormally increased muscle tone, and pseudobulbar syndrome. For the violation of a lower motor neuron lesion, the markers were as follows: peripheral paresis, fasciculations (muscle twitching), atrophy, and bulbar syndrome.

The functional status of the study participants was assessed using the revised version of the ALS Functional Rating Scale (ALS FRS-R) (28). Their nutritional status was assessed by measuring anthropometric and biochemical parameters. Anthropometric measurements included body weight (kg), body mass index (BMI), mid-upper arm circumference (MAC), mid-arm muscle circumference (MAMC), and triceps skinfold (TS). BMI was calculated using the following formula: BMI = m/h^2^, where *m* represents body weight (kg) and *h* is the height (cm^2^). For MAMC, the calculation formula was as follows: MAMC (cm) = MAC (cm) – (0.314 * TS (mm)).

The prognostic nutritional index (PNI) was determined. PNI was calculated as 100–1.5* serum albumin (g/l) − 1* MAC (cm) and then classified into four groups: eutrophy (≤20 points), first-degree (mild) malnutrition (21–30 points), second-degree (moderate) malnutrition (31–40 points), and third-degree (severe) malnutrition (>40 points).

All patients underwent the following laboratory tests: complete blood count (CBC), urinalysis, coprological examination, ionogram, and coagulation test. Data on the following biochemical parameters were collected: blood glucose, blood urea, serum creatinine, transferrin, iron, total protein, albumin, total bilirubin, thymol, liver transaminase activity, and lipid profile. The nutrition status was evaluated by measuring the following parameters in the blood: total lymphocyte count, total protein, albumin, urea, creatinine, transferrin, and iron. The amount of transferrin was determined by immunonephelometric analysis using a standard TRSF2/Transferrin Gen.2 kit (Roche Diagnostics GmbH, Germany) on a Cobas C311 analyzer (Germany). Patients also underwent MRI of the brain and spinal cord, spirometry, electrocardiography (ECG), and ENMG.

During our investigation, patients afflicted with amyotrophic lateral sclerosis (ALS) underwent systematic clinical assessment and monitoring. All patients were enrolled in the study at its inception and underwent evaluations of their baseline functional states, including the utilization of the ALS Functional Rating Scale (ALSFRS) and other clinical parameters, to establish fundamental disease status information.

Patients routinely visited our medical center for clinical assessments and laboratory analyses. The intervals between these visits and the execution of laboratory investigations were contingent upon the individual requirements of each patient and the progression of their respective conditions. We aimed to guarantee optimal medical care and consistent monitoring of patients’ conditions, thus meticulously tracing the progression of the disease.

Regarding pharmacological interventions and ALS treatment, throughout the study, we examined the comprehensive clinical profile of each patient, encompassing data about administered pharmaceutical agents and medications employed in the treatment of ALS.

Statistical analysis was performed in SPSS 13 and Microsoft Excel 2013 (Microsoft, USA). Data were compared using the Mann–Whitney U-test. Differences were considered statistically significant at a value of *p* <0.05. Fisher’s exact test was used for qualitative comparison. The correlation analysis was carried out using the Spearman’s correlation method.

This study respects ethical standards and principles, including the Declaration of Helsinki on Human Rights (1964), the Principles of the ICH GCP (1996), the Convention on Human Rights and Biomedicine (04.04.1997), and the EU Council Directive No. 609 (11.24.1986). The study protocol band the patient’s informed consent form were approved by the Biomedical Ethics Commission. In the study, ethical and moral norms were adhered to, ensuring that all acquired information is guaranteed to be anonymous and confidential. A consent agreement for participation in the research was signed with each of the patients.

## 3 Results

The amount of ALS diagnoses varied depending on the form of ALS. Some 17 (37.8%) patients were diagnosed as having cervicothoracic ALS. The lumbosacral ALS was present in 13 (28.9%) patients. The bulbar region was affected in 12 (26.7%) patients. Finally, the primary generalized and ‘high’ forms, as specified in Ovakim Khondkarian’s classification, were present in 2 (4.4%) patients and 1 (2.2%) patient, respectively. The mean time from disease onset to diagnosis was 8.20 ± 1.03 months.

Patients with ALS have significantly lower anthropometric parameters than practically healthy controls (Table 2, *p* < 0.05). Specifically, their body weight was 1.28 times lower (*p* < 0.05), and their BMI was lower by 1.30 times (*p* < 0.05). There were no statistically significant differences in patient height between groups (*p* > 0.05).

**Table 2:** Comparative anthropometric profiles for patients with ALS and practically healthy individuals, M ± m.

Variables	Practically healthy individuals (*n* = 30)	Patients with ALS (*n* = 45)
Weight, kg	67.53 ± 4.07	52.90 ± 2.85*
Height, cm	166.17 ± 5.29	167.24 ± 4.91
BMI, kg/m^2^	24.50 ± 1.03	18.97 ± 0.72*
TS, mm	12.60 ± 0.72	10.05 ± 0.48*
MAC, cm	28.58 ± 0.81	24.10 ± 0.53*
MAMC, cm	24.61 ± 0.48	20.94 ± 0.36*
PNI	1.72 ± 0.13	25.35 ± 1.60*

*Differences are statistically significant compared with practically healthy individuals (*р* < 0.05); TS, triceps skinfold; MAC, mid-upper arm circumference; MAMC, mid-arm muscle circumference; PNI, prognostic nutritional index.

Triceps skinfold measurements in patients with ALS had 1.25 times lower values than that in practically healthy individuals (*p* < 0.05). The same trend was observed with MAC and MAMC, whose values decreased by 15.7% (*p* < 0.05) and 14.9% (p < 0.05), respectively. Patients with ALS had 14.70 times (*p* < 0.05) higher PNI than practically healthy controls, showing a significant difference.

Based on biochemical findings, individuals diagnosed with ALS had 1.28 times lower levels of total protein in their blood than practically healthy controls (Table 3, *p* < 0.05). Their blood levels of albumin were also lower by 1.38 times (*p* < 0.05).

**Table 3:** Comparative biochemical profiles for patients with ALS and practically healthy individuals, M ± m.

Variables	Normal reference values for biochemical parameters	Practically healthy individuals (*n* = 30)	Patients with ALS (*n* = 45)
Total protein, g/L	66–83	77.80 ± 4.41	60.55 ± 2.38*
Albumin, g/L	35–52	46.49 ± 3.22	33.70 ± 2.03*
Total bilirubin, μmol/L	3.4–20.5	14.25 ± 1.06	13.42 ± 1.17
Urea, mmol/L	2.5–7.1	5.37 ± 0.50	3.09 ± 0.36*
Creatinine, μmol/L	44–97	70.91 ± 5.13	51.28 ± 4.42*
Total cholesterol, mmol/L	3.6–5.7	4.04 ± 0.39	3.55 ± 0.34
Potassium, mmol/L	3.5–5.1	4.20 ± 0.27	3.54 ± 0.31
Sodium, mmol/L	135–145	140.30 ± 0.65	138.69 ± 0.54
Calcium, mmol/L	2.15–2.55	2.38 ± 0.24	2.20 ± 0.15
Chlorine, mmol/L	98–107	104.76 ± 0.80	102.37 ± 0.69
Iron, μmol/L	10.7–28.3	20.68 ± 2.18	13.86 ± 2.53
Transferrin, g/L	2.0–3.6	2.32 ± 0.10	1.84 ± 0.12*
Total lymphocyte count, ×10^9^/L	1.0–4.0	2.86 ± 0.25	1.49 ± 0.11*

*Differences are statistically significant compared to practically healthy individuals (*р* < 0.05).

The table presents the lower reference values for normal parameters of biochemical indicators.

No statistically significant differences were found for total bilirubin, its fractions, and liver transaminase activity (*p* > 0.05). None of these findings were observed in blood electrolyte concentrations; however, there was a downward trend in the values of Na+, K+, Ca2+, and Cl− (*p* > 0.05).

As for biochemical parameters that characterize protein metabolism, patients with ALS had 1.74 times lower concentrations of urea and 1.38 times lower concentrations of creatinine in their blood than practically healthy controls; these differences were statistically significant (*p* < 0.05). Unlike the control group participants, patients with ALS experienced a reduction in total cholesterol (*p* > 0.05). Transferrin was significantly lower in patients with ALS than in control group participants (*p* < 0.05). The serum iron level was also lower, but the difference was not significant (*p* > 0.05). Patients with ALS had 1.90 times lower total lymphocyte count compared with practically healthy controls (*p* < 0.05). As for other blood parameters, no statistically significant differences were observed (*p* > 0.05).

The correlation analysis revealed a direct relationship of BMI with the following parameters: MAC (*r* = 0.51, *p* < 0.05), MAMC (*r* = 0.60, *p* < 0.05), TS (*r* = 0.56, *p* < 0.05), PNI (*r* = 0.82, *p* < 0.05), total protein (*r* = 0.22, *p* < 0.05), albumin (*r* = 0.27, *p* < 0.05), urea (*r* = 0.33, *p* < 0.05), creatinine (*r* = 0.30, *p* < 0.05), transferrin (*r* = 0.18, *p* < 0.05), and total lymphocyte count (*r* = 0.20, *p* < 0.05). PNI correlated with total protein (*r* = 0.53, *p* < 0.05), albumin (*r* = 0.87, *p* < 0.05), total cholesterol (*r* = 0.34, *p* < 0.05), transferrin (*r* = 0.40, *p* < 0.05), total lymphocyte count (*r* = 0.79, *p* < 0.05), urea (*r* = 0.37, *p* < 0.05), and creatinine (*r* = 0.32, *p* < 0.05). MAMC correlated with total protein (*r* = 0.20, *p* < 0.05), albumin (*r* = 0.22, *p* < 0.05), urea (*r* = 0.53, *p* < 0.05), and creatinine (*r* = 0.50, *p* < 0.05).

## 4 Discussion

Among the goals of this study was determining whether biochemical parameters may be potentially helpful in assessing the nutritional status of patients with ALS and whether they correlate with anthropometric measurements. The present findings revealed a significant decrease in anthropometric indicators (weight, BMI, MAC, MAMC, and TS) in patients with ALS compared with practically healthy controls. The mean values of BMI, MAC, MAMC, and TS correspond to mild malnutrition (Table 2). Another piece of evidence that suggests the presence of first-degree malnutrition in patients with ALS is a significant increase in PNI, which was found to be 14.0 times higher than that in controls.

However, assessing anthropometric indicators is not enough to determine the nutritional status of patients with ALS, for they only provide information about the peripheral reserves of protein. The visceral protein status is characterized by biochemical parameters, such as total protein, albumin, retinol-binding protein, and transferrin. The extent to which these indicators are reduced depends on nutritional deficiency (29, 30).

This study examined the common biochemical indicators (Table 3). The blood level of the total protein is indicative of protein metabolism. In this study, patients with ALS had significantly lower total protein in their blood than controls, which suggested the presence of mild malnutrition. Note that total protein is an insensitive marker, for it often gives false negative results, likely due to an increase in globulin or dehydration. A more reliable indicator for assessing nutritional status is albumin (29). In this study, patients with ALS had lower albumin in their blood compared with control participants, which is typical for first-degree malnutrition. However, there are two factors that can affect the information capacity of albumin: (1) a transfer of interstitial albumin into the intravascular pool and (2) a relatively long half-life of albumin. In addition, albumin levels in the liver could be low due to a decrease in protein synthesis. There were no cases of liver damage observed in this study; total bilirubin levels and the activities of hepatic transaminases were within the normal range and did not differ significantly between groups.

Among markers of protein catabolism are urea and creatinine, as confirmed by the correlation between these two markers and BMI in patients with ALS. Both indicators showed lower values in patients with ALS, suggesting first-degree malnutrition. Note that the information capacity of these indicators depends on the nature of nutrition and on whether the patient’s kidney function is impaired or not.

Patients with ALS had lower levels of transferrin in their blood compared with control participants. The presence of hepatic and (or) kidney failure, however, can affect the reliability of this indicator, which was not the case here. Patients with ALS also had significantly lower lymphocyte count compared with controls. The value of the total lymphocyte count observed in patients with ALS suggests mild malnutrition.

The present findings are comparable with the results of similar studies (31–33). An indicative example is a study conducted on 193 patients with ALS in South Korea (31). Researchers have observed a reduction in body mass index (BMI), as well as some deterioration in nutritional status indicators, along with decreased levels of total protein, albumin, urea, and creatinine in the blood of patients with amyotrophic lateral sclerosis (ALS). Furthermore, investigations have revealed correlations between BMI and the levels of albumin (*r* = 0.150, *p* < 0.010), creatinine (*r* = 0.206, *p* < 0.01), and urea (*r* = 0.239, *p* < 0.01). Researchers have also noted an association between the geriatric nutritional risk index and the levels of total protein (*r* = 0.485, *p* < 0.01), albumin (*r* = 0.737, *p* < 0.01), and total cholesterol (*r* = 0.218, *p* < 0.01) (29).

Based on previous research (31–33), it can be argued that there is no single biochemical indicator that could become a universal and accurate marker for assessing the nutritional status of patients with ALS. Some studies highlight the beneficial effect of high-calorie nutrition on life duration and nutritional status, even though no significant effect on blood lipid levels was determined (34). As the COVID-19 pandemic began to spread, the need to develop new methods for managing patients with ALS became more evident (35–37). Furthermore, the lockdown was reported to impact caregivers more than the patients with ALS (38). An important prognostic indicator of ALS is weight loss, primarily through muscle and adipose tissues (39). The percutaneous endoscopic gastrostomy is considered the safest nutritional method (40); however, since the main cause behind weight loss is malnutrition, further exploration of diet options is needed (41–45). While we acknowledge the availability of direct measures such as body mass index (BMI) for assessing the nutritional status of patients with ALS, the inclusion of biochemical parameters in our study serves a specific purpose. Biochemical markers, such as total protein, albumin, urea, creatinine, transferrin, and total lymphocyte count, offer insights into the intricate metabolic and immunological aspects of malnutrition.

It is crucial to recognize that ALS is a multifaceted disease with diverse effects on the body’s physiology. While BMI provides a straightforward measure of body weight relative to height, it may not capture the subtleties of malnutrition that occur at the cellular and molecular levels. Biochemical parameters, on the other hand, enable us to delve deeper into the dynamic changes occurring within the patient’s body.

These biochemical markers can help identify early signs of nutritional deficiencies, track disease progression, and even guide targeted interventions. For instance, alterations in total protein and albumin levels reflect changes in protein metabolism and visceral protein status, shedding light on potential nutritional deficits. Additionally, the urea and creatinine levels can indicate shifts in protein catabolism and kidney function, both of which are pertinent to patients with ALS.

While our study demonstrates correlations between anthropometric and biochemical parameters, we emphasize that these two approaches should be viewed as complementary rather than redundant. Together, they provide a more comprehensive understanding of the nutritional landscape in ALS. The integration of biochemical parameters enhances our ability to tailor nutritional strategies to the specific needs of each patient, potentially improving their overall care and quality of life.

In conclusion, the incorporation of biochemical parameters alongside traditional anthropometric measures enriches our evaluation of the nutritional status of patients with ALS. This complementary approach empowers healthcare professionals to address the multifaceted challenges posed by ALS, offering a more holistic perspective on patient care. Further research can explore the clinical applications of these biochemical markers and their potential benefits in guiding personalized nutritional interventions.

It is important to evaluate biochemical parameters comprehensively. By assessing the reduction in the blood levels of total protein, albumin, urea, creatinine, transferrin, and total lymphocyte count, one can identify the extent to which the nutritional status of the ALS patient has deteriorated, as evidenced by the presence of direct correlations between these biochemical parameters, BMI, and PNI. Future research can focus on developing effective metabolic treatments for patients with ALS with nutritional status disorders.

## 5 Conclusion

This study provides crucial data on the nutritional status of patients with amyotrophic lateral sclerosis (ALS). Identified low values of anthropometric and biochemical parameters, such as BMI, TS, MAMC, total protein, albumin, urea, creatinine, transferrin, and overall lymphocyte count, indicate the presence of first-degree malnutrition in ALS patients. Positive correlations between anthropometric and biochemical parameters highlight the importance of utilizing biochemical indicators for assessing nutritional status in this condition. These findings can be valuable for practicing physicians specializing in the care of ALS patients in developing more effective strategies for managing nutritional status and improving the quality of life for these individuals.

The results of this study provide important insights into understanding the nutritional status of ALS patients. However, despite valuable conclusions, certain limitations of this research need consideration. Limitations include a restricted sample size and a limited study period. Future research efforts may expand the sample size and extend the observation period to obtain a more comprehensive understanding of the dynamics of nutritional status in ALS patients. Additionally, the possibility of incorporating additional factors, such as lifestyle and physical activity levels, should be explored for a more in-depth analysis of their impact on nutritional status.

## Data availability statement

The raw data supporting the conclusions of this article will be made available by the authors, without undue reservation.

## Ethics statement

The studies involving humans were approved by the ethics committee of Medical University Sofia. The studies were conducted in accordance with the local legislation and institutional requirements. The participants provided their written informed consent to participate in this study.

## Author contributions

DM: Conceptualization, Formal analysis, Investigation, Methodology, Project administration, Supervision, Writing – review & editing. NM: Data curation, Funding acquisition, Resources, Software, Validation, Visualization, Writing – original draft.
